# Preparation and Performance Verification of a Solid Slow-Release Carbon Source Material for Deep Nitrogen Removal in Urban Tailwater

**DOI:** 10.3390/molecules29092031

**Published:** 2024-04-28

**Authors:** Zhang Luo, Hongtao Shi, Hanghang Lyu, Hang Shi, Bo Liu

**Affiliations:** 1 China Railway Engineering Services Co., Ltd., Chengdu 610083, China; luozhang@cresc.cn (Z.L.);; 2 China Construction Eighth Engineering Division Co., Ltd., Shanghai 200135, China; 3 Yalong River Hydropower Development Company, Chengdu 610051, China; 4 State Key Laboratory of Pollution Control and Resource Reuse, School of the Environment, Nanjing University, #163, Xianlin Avenue, Nanjing 210023, China

**Keywords:** slow-release carbon source, slow-release coefficient, denitrification, low carbon-to-nitrogen ratio wastewater

## Abstract

Urban tailwater typically has a low carbon-to-nitrogen ratio and adding external carbon sources can effectively improve the denitrification performance of wastewater. However, it is difficult to determine the dosage of additional carbon sources, leading to insufficient or excessive addition. Therefore, it is necessary to prepare solid slow-release carbon source (SRC) materials to solve the difficulty in determining the dosage of carbon sources. This study selected two SRCs of slow-release carbon source 1 (SRC1) and slow-release carbon source 2 (SRC2), with good slow-release performance after static carbon release and batch experiments. The composition of SRC1 was: hydroxypropyl methylcellulose/disodium fumarate/polyhydroxy alkanoate (HPMC/DF/PHA) at a ratio of 3:2:4, with an Fe_3_O_4_ mass fraction of 3%. The composition of SRC2 was: HPMC/DF/PHA with a ratio of 1:1:1 and an Fe_3_O_4_ mass fraction of 3%. The fitted equations of carbon release curves of SRC1 and SRC2 were *y* = 61.91 + 7190.24e^−0.37*t*^ and *y* = 47.92 + 8770.42e^−0.43*t*^, respectively. The surfaces of SRC1 and SRC2 had a loose and porous morphological structure, which could increase the specific surface area of materials and be more conducive to the adhesion and metabolism of microorganisms. The experimental nitrogen removal by denitrification with SRCs showed that when the initial total nitrogen concentration was 40.00 mg/L, the nitrate nitrogen (NO_3_^−^-N) concentrations of the SRC1 and SRC2 groups on the 10th day were 2.57 and 2.66 mg/L, respectively. On the 20th day, the NO_3_^−^-N concentrations of the SRC1 and SRC2 groups were 1.67 and 2.16 mg/L, respectively, corresponding to removal efficiencies of 95.83% and 94.60%, respectively. The experimental results indicated that SRCs had a good nitrogen removal effect. Developing these kinds of materials can provide a feasible way to overcome the difficulty in determining the dosage of carbon sources in the process of heterotrophic denitrification.

## 1. Introduction

Due to the incomplete removal of total nitrogen (TN) in sewage treatment and the continuous accumulation of nitrogen in water, nitrate pollution of water bodies has become one of the important environmental problems at present [1,2]. The traditional denitrification technology mainly involves the nitrification and denitrification process, and organic carbon sources (OCs) are the core base materials of this process. Consequently, when the C/N ratio in the wastewater is low, it is usually necessary to add appropriate OCs into the wastewater to achieve adequate denitrification and ensure the nitrogen removal effect. Common OCs include methanol and acetic acid [3].

In the dosing process, the liquid carbon source tends to be thoroughly mixed with the sewage upon dosing. It is difficult to dynamically adjust the dosage according to the change in sewage quality, resulting in challenges such as high costs, cumbersome operations, maintenance, and challenges determining the dosage of carbon sources. Moreover, an excessive dosage of carbon sources can cause secondary pollution. Hence, researchers are attempting to develop solid slow-release carbon sources (SRCs) that can continuously and stably release chemical oxygen demand (COD) within certain concentration ranges, avoid secondary pollution, and simplify the dosing procedure.

At present, the SRCs mainly include synthetic materials and natural materials, such as wood chips, straw, corncob, wheat straw, and peanut shells [4,5,6,7]. Studies have shown that carbon-based microspheres, prepared by embedding corncob with a particle size less than 0.125 mm into sodium alginate, have a good carbon release performance and that the carbon release rate of microspheres can be controlled by changing the number of microspheres [8]. Natural materials are feasible as OCs, but fewer can be effectively utilized. Moreover, using these carbon sources usually involves the generation of by-products, and the removal efficiency is poor when these carbon sources are used to deal with high concentrations of nitrate nitrogen (NO_3_^−^-N) [5,9,10]. In contrast, synthetic materials are usually polymers with good biodegradabilities, such as polyhydroxy alkanoate (PHA), poly-3-hydroxybutyrate-co-valerate (PHBV), poly-3-hydroxybutyrate (PHB), polybutylene succinate (PBS), polycaprolactone (PCL), and polylactic acid (PLA) [11,12,13]. They are stable in nature, have low COD releases, and can be degraded by microorganisms; however, the disadvantage is the relatively high cost. A composite SRC, with a diameter of 0.3 cm and a length of 2 cm, was prepared using PLA and starch, and denitrification experiments were conducted on groundwater contaminated with NO_3_^−^-N [14]. Results showed that the NO_3_^−^-N removal efficiency reached 99%. In another study, microfibers and solid carbon sources were used for in situ ecological restoration of wetlands [15]. Compared with traditional nitrogen removal treatment, the total nitrogen removal efficiency increased from 20.6 ± 4.0% to 90.4 ± 2.7%, which increased denitrification efficiency in low C/N ratio environments. Lignin contains a high proportion of carbohydrates [16,17], and a sawdust–skeleton composite substrate (with wood, slag, and gravel as the substrate) was constructed as an SRC in a baffle-underflow artificial wetland [18]. The results showed that the removal efficiency of ammonia nitrogen and total nitrogen reached 37.5~85% and 57.4~86%, respectively, proving that combining these SRCs was feasible.

Disodium fumarate (DF) was approximately 80% cheaper than lactate [19] and has a better denitrification rate compared to sodium propionate, sodium formate, and lactic acid for carbon sources [20]. However, few studies have been conducted on DF-based slow-release carbon sources. As mentioned above, the preparation of solid SRCs with large release amounts, long release time, good filling performance, and good nitrogen removal effect is the key to improving the NO_3_^−^-N removal efficiency. In this study, DF, PHA, hydroxypropyl methylcellulose (HPMC), and magnet powder were used to prepare SRCs. The carbon release characteristics and nitrogen removal performance of the SRCs, with different ratios of raw materials, were investigated through experiments. This study can provide theoretical support for the application of SRC1 (HPMC/DF/PHA: 3:2:4) and SRC2 (HPMC/DF/PHA: 1:1:1) in wastewater treatment.

## 2. Results and Discussion

### 2.1. Static Release Rules of Slow-Release Carbon Sources

**Figure 1:**
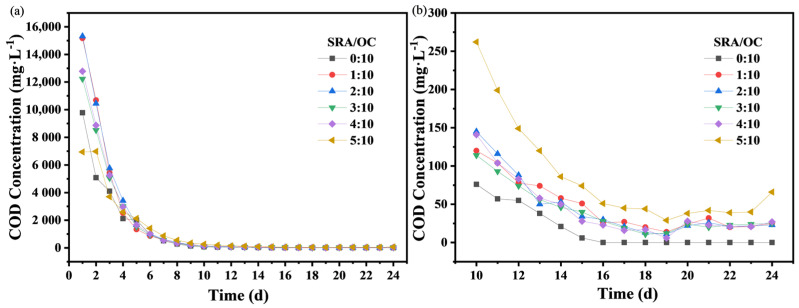
Influence of the dosage of slow-release agents (SRAs) on carbon release rate, (**a**): 0–24 days, (**b**): 10–24 days.

The static carbon release experiment was designed to explore the influence of different carbon source proportions, the SRA dosage, and carbon source types on the SRCs. The influence of the dosage of HPMC as an SRA on the carbon release rate was investigated by six batch experiments. The experimental results are shown in Figure 1.

In the initial stage of carbon release, a large amount of carbon sources was released, and the carbon release curves show a steep downward trend. With the increase in time, the carbon release curves flattened out, and the COD release rate slowed down. At the initial stage of carbon release, most of the COD comes from the surface of the carbon source. When the surface carbon source was released to the peak value, the main release zone shrank to the interior of the material, resulting in a longer diffusion path and a gradually decreasing concentration gradient. When no SRA was added, the carbon release process of the material was very rapid, and the carbon source release tended to zero on the 16th day. With the increased proportion of SRA, the slow-release effect was gradually enhanced, and the released carbon source remained stable after the 10th day. After the 16th day, the carbon source release concentrations of the materials with SRA/OC mass ratios of 1:10, 2:10, 3:10, and 4:10 ranged from 11 to 30 mg/L, and the average concentrations were 23.20, 21.00, 20.67, and 20.22 mg/L, respectively. The carbon source release concentration of the material with the SRA/OC mass ratio of 5:10 ranged from 29 to 66 mg/L, and the average concentration was 43.78 mg/L.

According to Table 1, the correlation coefficients S^2^ of the carbon release curves and the fitted exponential curve equations were greater than 0.99. Comparing the decay coefficients *k* of the six curves showed that the value of *k* decreased with the increase in the HPMC dosage, indicating an improved slow-release performance of the material. When the mass ratio of the SRA to organic carbon source was 5:10, *k* had a minimum value of 0.33, indicating an optimal slow-release performance. This proved that adding HPMC was conducive to the slow release of OCs in the material. Considering both the slow-release performance and the effective utilization rate of the material, an SRA/OC mass ratio of 5:10 was selected for the preparation of the SRCs.

**Figure 2:**
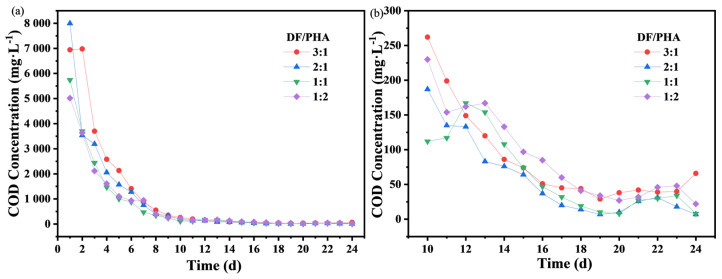
Influence of DF/PHA ratios in the carbon source on the carbon release rate, (**a**): 0–24 days, (**b**): 10–24 days.

To prepare SRCs, DF and PHA were selected as OCs. To explore the influence of the ratio of DF/PHA on the carbon release rate of SRCs, five batches of experiments were conducted. The experimental results are shown in Figure 2.

In the batch experiments, it was found that when DF/PHA was 4:1, the material structure was soaked to a loose state during the static carbon release, and the turbidity of the leaching solution was high, which did not meet the application requirements. This phenomenon also proved that PHA can act as a supporting skeleton structure to maintain the stability of the material in SRCs.

Figure 2 shows that in the earlier stage of carbon release, the higher the DF/PHA ratio, the faster the release rate of carbon sources. In the beginning, the initial COD concentrations were high due to the release of readily decomposable carbohydrates and the rapid degradation of water-soluble substances [21,22]. At 0–6 days, the initial carbon release rates of SRCs with DF/PHA ratios of 3:1 and 2:1 were considerably higher than those of SRCs with DF/PHA ratios of 1:1 and 1:2. Then, with the easily decomposable components of the structure within the SRCs further decomposing, dissolving, and releasing into the water, the hardly decomposable substances accumulated; hence, COD release was unstable, and the concentration suddenly increased between day 11 and 14, and ultimately achieved a balance state after the 14th day [23,24]. The released carbon concentrations for DF/PHA ratios 3:1 and 1:2 were relatively high. After the 16th day, the carbon source release concentrations of the materials with DF/PHA ratios of 3:1, 2:1, 1:1, and 1:2 ranged from 7 to 85 mg/L, and the average concentrations for the materials with DF/PHA ratios of 3:1, 2:1, 1:1, and 1:2 were 43.78, 18.89, 23.89, and 43.89 mg/L, respectively.

To sum up, when DF and PHA were combined as OCs, the initial carbon release of the material was high if the DF/PHA ratio was high. When the DF/PHA ratio was low, the long-term carbon release rate of the material remained stable.

According to Table 2, the carbon release curves of the SRCs still had good fitting relationships with the exponential curves. The correlation coefficients for the fitting curves, corresponding to the DF/PHA ratios of 1:2 and 1:1, were greater than 0.99. In addition, with the decrease in the DF/PHA ratio, the decay coefficient, *k*, first increased and then decreased. When the DF/PHA ratio was 2:1, *k* reached an extreme value of 0.46, indicating the worst slow-release performance. Overall, the slow-release performance of the carbon source was the best when the DF/PHA ratio was 1:2 or 1:1.

**Figure 3:**
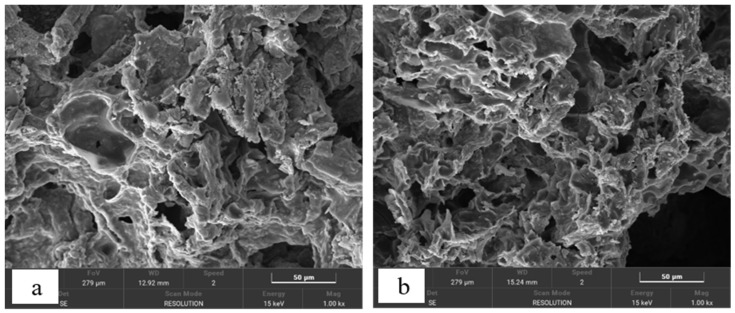
Scanning electron microscopy spectroscopy images of the slow-release carbon sources: (**a**) SRC1 and (**b**) SRC2.

The surface of the SRCs was loose and porous, with a large specific surface area, which was more conducive to the adhesion and growth of microorganisms (Figure 3). In both SRC1 and SRC2, the mass percentage of C was around 50% (Table 3). In addition, the weight percentage of Na in SRC1 was significantly lower than in SRC2. This can be attributed to the low proportion of DF in SRC1. In SRC1 and SRC2, the weight percentage of Fe was significantly higher than the theoretical value, and the total confidence coefficient was also high, indicating that the distribution of Fe on the surface of the filler was insufficient.

### 2.2. Influence of SRC on the Effect of Nitrogen Removal by Denitrification

**Figure 4:**
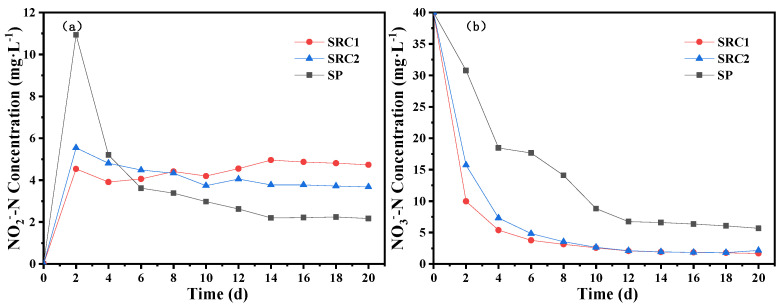
Nitrogen removal effects of slow-release carbon source 1 (SRC1), slow-release carbon source 2 (SRC2), and sulfur particle (SP) as a function of (**a**) NO_2_^−^-N and (**b**) NO_3_^−^-N concentrations.

In the batch experiments, each experimental group had different degrees of NO_2_^−^-N accumulation (Figure 4a). At the beginning of the experiment, a relatively high concentration of NO_2_^−^-N was accumulated in the SP group, reaching a peak value of 10.93 mg/L on the second day, whereas the concentrations of NO_2_^−^-N in the SRC1 and SRC2 groups were only 4.54 and 5.54 mg/L, respectively. With the extension of the reaction, the concentrations of NO_2_^−^-N in the SRC2 and SP groups decreased gradually, while that in the SRC1 group increased slightly. On the 14th day, the concentration of NO_2_^−^-N in the SRC1 group reached a peak value of 4.96 mg/L, whereas those in the SRC2 and SP groups were 3.77 and 2.20 mg/L, respectively, which were lower than that in the SRC1 group.

**Figure 5:**
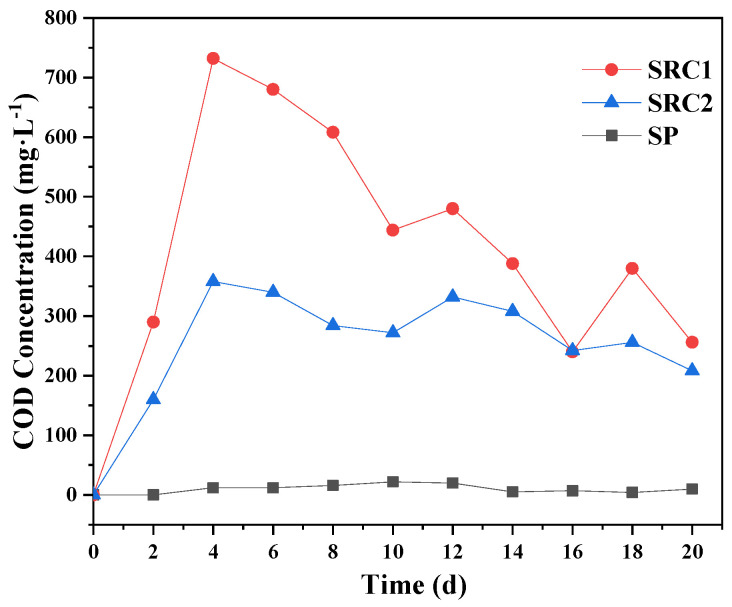
Changes in the chemical oxygen demand (COD) value for each group in the batch experiments.

In the batch experiments, the concentration of NO_3_^−^-N decreased rapidly at the initial stage of the reaction, and the NO_3_^−^-N removal efficiency in the SRC1 and SRC2 groups was considerably higher than that in the SP group. With the extension of the reaction, the NO_3_^−^-N removal efficiency of each group decreased slightly. On the second day, the concentration of NO_3_^−^-N in the SP group was 30.78 mg/L, which was much higher than 9.96 mg/L in the SRC1 group or 15.72 mg/L in the SRC2 group. On the 10th day, the concentrations of NO_3_^−^-N in the SRC1 and SRC2 groups were 2.57 and 2.66 mg/L, respectively, which was lower than that in the SP group (8.80 mg/L). On the 20th day, the concentrations of NO_3_^−^-N in SRC1, SRC2, and SP groups were 1.67, 2.16, and 5.68 mg/L, respectively, and the NO_3_^−^-N removal efficiencies were 95.83%, 94.60%, and 85.79%, respectively, indicating the sufficient nitrogen removal effect. The results verified that SRC1 and SRC2 had good bioavailability and can ensure a smooth heterotrophic denitrification process. The sulfur autotrophic denitrification in the SP group had a slower denitrification rate; however, the accumulated concentration of NO_2_^−^-N was lower. The denitrification rates in the SRC1 and SRC2 groups were faster; the NO_3_^−^-N removal efficiency can reach about 95%, and the accumulation of nitrite was also low. On the 14th day, the concentrations of NO_2_^−^-N in the SP, SRC1, and SRC2 groups were 2.20, 4.96, and 3.77 mg/L, respectively. At the initial stage of the experiment, the COD concentrations in the SRC1 and SRC2 groups increased rapidly and reached maximum values of 732 and 358 mg/L, respectively, on the fourth day. Then, the COD concentrations gradually decreased and fluctuated within the range of 200–300 mg/L. The carbon release amount of the SRC1 group was higher than that of the SRC2 and S groups (Figure 5), and the corresponding nitrogen removal effect of the SRC1 group was also the best. In addition, the SP group was observed to contain low concentrations of COD, which could be attributed to the effect of microbial activity.

**Figure 6:**
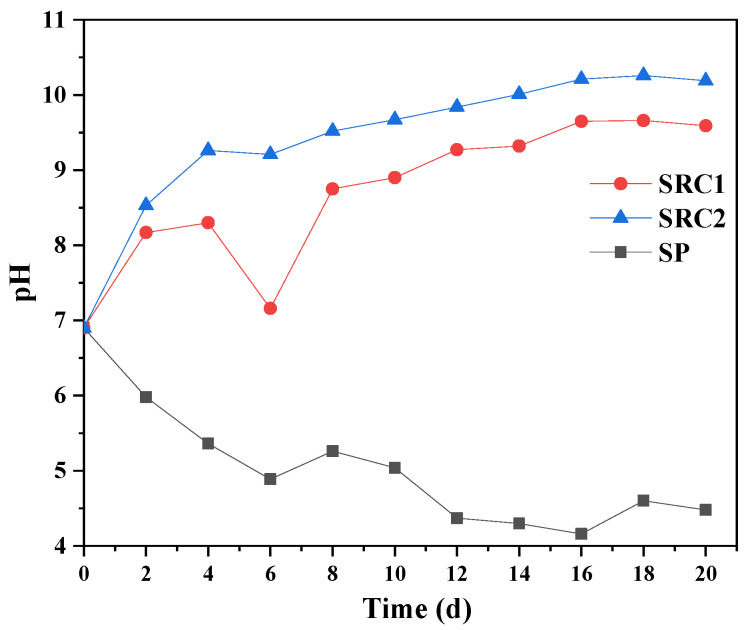
Changes in pH values for each group in the batch experiments.

The pH of the SRC2 group was always higher than that of the SRC1 group, and the two groups reached maximum values of 10.26 and 9.66 on the 18th day, respectively (Figure 6). The pH value of the SP group gradually decreased and reached the extreme value of 4.16 on the 16th day. The SO_4_^2−^ concentration of the SP group reached the extreme value of 160 mg/L on the 16th day, which was consistent with the occurrence time of the extreme value of pH value.

**Figure 7:**
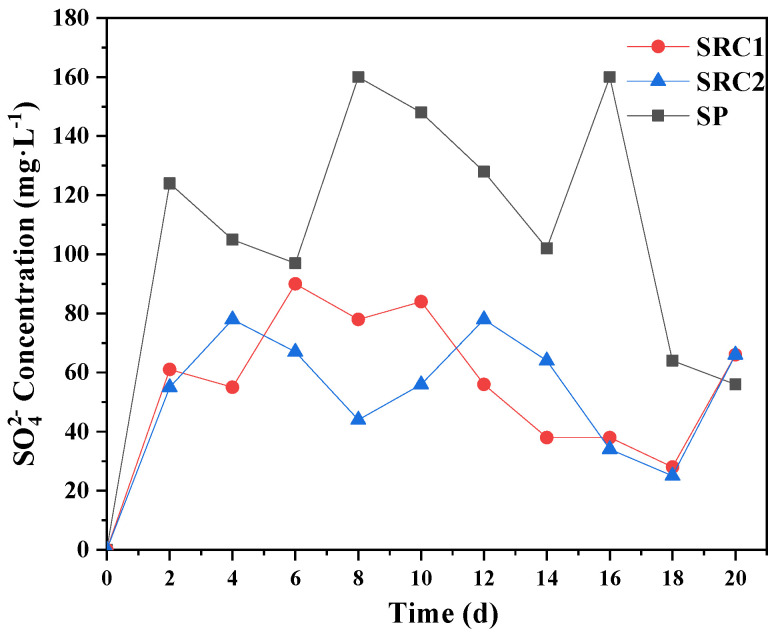
Changes in SO_4_^2−^ concentration for each group in the batch experiments.

The comparison of experimental results in the SRC1, SRC2, and SP groups showed that the SRCs can be successfully applied to biological nitrogen removal. The batch experiments indicated that when only sulfur autotrophic denitrification or heterotrophic denitrification was involved, the pH value may be too low or too high, and the SO_4_^2−^ or COD concentration in the effluent water may be too high (Figure 5 and Figure 7). Therefore, sulfur autotrophic denitrification and heterotrophic denitrification should be coupled to construct a deep denitrification reaction system, which was conducive to the equilibrium of microorganisms in the system and guaranteed the effluent effect.

## 3. Materials and Methods

### 3.1. Preparation of SRCs

The preparation of SRCs requires three kinds of raw materials, including OCs, viscous materials, and slow-release agents (SRAs). After comparing various OCs, a combination of DF and PHA was selected as the carbon source of the targeted material. This combination has good biodegradability and can avoid secondary pollution. The HPMC was selected as the SRA [25,26]. In addition, Fe_3_O_4_ powder was added as one raw material for the preparation of the SRCs, which can adjust the material density and prevent the floating of the SRCs. Moreover, the weak magnetism of Fe_3_O_4_ powder is conducive to the adhesion of various materials. It enhances the biological activity of activated sludge to a certain extent [27], which is conducive to improving the nitrogen removal efficiency of the denitrification filter [28,29].

The main preparation procedure for the SRCs refers to Supplementary Information.

### 3.2. Static Carbon Release Experiment and Batch Carbon Release Experiments

Six batch experiments were conducted with the mass ratios of the SRA/OC of 0:10, 1:10, 2:10, 3:10, 4:10, and 5:10, respectively, to investigate the influence of the dosage of HPMC as an SRA on the carbon release rate. Five batches of experiments were conducted with different DF/PHA mass ratios of 4:1, 3:1, 2:1, 1:1, and 1:2, respectively, under the premise of the SRA/OC mass ratio of 5:10, to investigate the influence of the ratio of DF/PHA on the carbon release rate of SRCs. SRCs (each 2.50 g) were rinsed with 20 mL of pure water to remove impurities and placed in 50 mL sample centrifuge tubes, followed by the addition of distilled water (40 mL) [30,31]. The centrifugal tubes were sealed and placed at room temperature, 25 °C. Samples (20 mL) were extracted every other day and immediately replenished with the same volume. The samples were oscillated and centrifuged; the supernatant was taken for COD measurement.

SRCs and sulfur particles (SPs) were selected for simulated wastewater denitrification batch experiments. Then, 2.50 g of fillers, 5 mL denitrifying bacteria solution, and 40 mL simulated wastewater were put into 50 mL serum flasks and inoculated [32]. The simulated wastewater had a NO_3_^−^-N concentration of 40.00 mg/L. The fillers were SPs and SRCs. SPs consist of industrial sulfur, with purity ≥99.6%. After washing with distilled water, SPs were soaked in 75% ethanol for 10 h, then washed with distilled water and dried for use.

### 3.3. SEM-EDS Analyses

In this study, SPs and SRCs were used as the reactor fillers. The surface morphology and microorganism morphology on the surface of the fillers were observed and analyzed by scanning electron microscopy and energy-dispersive X-ray spectroscopy (SEM-EDS) (Zeiss-Supra 55, Caise, Jena, Germany) [33]. And the specific procedure for the sample pretreatment is in the Supplementary Information.

### 3.4. Analysis Items and Methods

The monitoring and analysis methods are referred to as the “Monitoring and Analysis methods of Water and Wastewater” (4th Edition). The pH value was determined by a portable pH meter, the concentrations of NO_3_^−^-N, NO_2_^−^-N, and SO_4_^2−^ were determined by an ion chromatograph (Dionex ICS-1100, Thermo, Waltham, USA), and the COD value was determined according to the national standard method.

### 3.5. Data Analysis

To compare the carbon release rates of the SRCs, the carbon release process was fitted in the form of an exponential function to analyze the carbon release rules. The basic form of the exponentially fitted equation is:

(1) $$y=y_{0}+Ae^{-kt}$$

where y represents the concentration of COD_Cr_ in the leaching solution, mg/L; *t* is the release time, d; *k* represents the carbon release decay coefficient, d^−1^; *y*_0_ and *A* are constants.

## 4. Conclusions

The optimal SRCs, SRC1 and SRC2, were prepared by screening SRA dosage, carbon source types, and ratios through static release and batch experiments, and their denitrification performance was verified. SRCs were stable in the release of organic carbon from water and biodegradable, making them suitable as an external carbon source for biological nitrogen removal as well as a microbial carrier. The results of SEM-EDS analysis of SRCs further suggested that they favored microbial adhesion and metabolism. Meanwhile, the nitrogen removal efficiency of SRC1 and SRC2 was 95.83% and 94.60% on the 20th day, respectively, which further verified the feasibility of the SRC preparation. Thus, the relatively cheaper DF-based SRC will have good prospects for application.

## Figures and Tables

**Table 1:** Fitted equations of carbon release decay curves at different dosages of slow-release agents (SRAs).

Mass Ratio of SRA/OC	Fitted Curve Equations	Carbon Release Decay Coefficient *k*	S^2^
0:10	y = 4.22 + 15,178.31e^−0.47t^	0.47	0.989
1:10	y = −28.11 + 26,711.89e^−0.53t^	0.53	0.990
2:10	y = −31.42 + 25,860.30e^−0.50t^	0.50	0.996
3:10	y = −35.10 + 20,111.55e^−0.47t^	0.47	0.996
4:10	y = −31.82 + 21,066.04e^−0.47t^	0.47	0.996
5:10	y = −38.40 + 10,687.64e^−0.33t^	0.33	0.969

**Table 2:** Fitted equations of carbon release decay curves at different DF/PHA ratios in carbon source.

DF/PHA Ratio	Fitted Curve Equations	Carbon Release Decay Coefficient *k*	S^2^
4:1	—	—	—
3:1	y = −38.40 + 10,687.64e^−0.33t^	0.33	0.969
2:1	y = 83.67 + 11,689.13e^−0.46t^	0.46	0.971
1:1	y = 47.92 + 8770.42e^−0.43t^	0.43	0.998
1:2	y = 61.91 + 7190.24e^−0.37t^	0.37	0.992

**Table 3:** Energy-dispersive X-ray analysis results from slow-release carbon source surface.

	SRC1		SRC2	
Element	Weight Percentage	Total Confidence Coefficient	Weight Percentage	Total Confidence Coefficient
C	50.43	0.27	49.66	0.22
O	26.90	0.22	25.56	0.18
Na	3.74	0.07	6.62	0.07
Si	9.51	0.09	10.91	0.08
Fe	9.42	0.22	7.26	0.15
Total	100.00		100.00	

## Data Availability

Data are contained within the article and Supplementary Materials.

## Supplementary Materials

The following supporting information can be downloaded at: https://www.mdpi.com/article/10.3390/molecules29092031/s1.
