# Supplementary material for: Preparation and Performance Verification of a Solid Slow-Release Carbon Source Material for Deep Nitrogen Removal in Urban Tailwater
Source: Molecules. 2024 Apr 28;29(9):2031. doi: 10.3390/molecules29092031 (PMC11085913; doi:10.3390/molecules29092031)
Supplement: Supplementary file 1 [file molecules-29-02031-s001.zip › molecules-2958229-supplementary.pdf]

1. Preparation of SRCs

Step 1 The DF, PHA, and HPMC were weighed separately and mixed evenly, and the mass ratio of DF, PHA, and HPMC was (20~40) : (30~50) : (20~30).

Step 2 Based on reports by Yeum et al. [24], Fe_3O_4 powder with a mass fraction of 3% was added to the above mixture in Step 1 and then mixed evenly.

Step 3 Sodium silicate solution, with a mass concentration of 50%, was added to the evenly mixed mixture in Step 2 and then stirred evenly. The mass ratio of the sodium silicate solution to the evenly mixed mixture in Step 2 was 1:1 ~ 1:2.

Step 4 The mixture in Step 3 was placed in an oven and sintered at a high temperature of 260°C for 2 h, and then cooled to room temperature to obtain the SRC.

Step 5 The obtained SRC was broken and screened, and the particle size of the carbon source material for subsequent experiments was 0.5~0.8 cm.

2. SEM-EDS

(1) The filler was collected to be analyzed and placed in a centrifugal tube. The filler was cleaned with deionized water three to four times, and the supernatant was discarded.

(2) A phosphate buffer of 0.1 M containing 2.5% glutaraldehyde (pH = 7.1) was prepared in advance, and the sample was soaked in the phosphate buffer, and placed in a refrigerator at 4℃ for 12 h to achieve fixation.

(3) After fixation, the sample was soaked in the phosphate buffer for 10 min, and then, the sample was cleaned with 25%, 50%, and 75% ethanol every 10 min, successively; finally, the sample was soaked twice (10 min each time) in 100%

ethanol to complete the dehydration operation.

(4) The dehydrated sample was soaked twice (5 min each time) in hexamethyldisilane, and then placed in a fume hood to air dry for at least 2 h.

(5) Gold was sprayed on the dried sample for analysis measurements.
